# Structures of the portal vertex reveal essential protein-protein interactions for Herpesvirus assembly and maturation

**DOI:** 10.1007/s13238-020-00711-z

**Published:** 2020-04-13

**Authors:** Nan Wang, Wenyuan Chen, Ling Zhu, Dongjie Zhu, Rui Feng, Jialing Wang, Bin Zhu, Xinzheng Zhang, Xiaoqing Chen, Xianjie Liu, Runbin Yan, Dongyao Ni, Grace Guoying Zhou, Hongrong Liu, Zihe Rao, Xiangxi Wang

**Affiliations:** 1 CAS Key Laboratory of Infection and Immunity, National Laboratory of Macromolecules, Institute of Biophysics, Chinese Academy of Sciences, Beijing, 100101 China; 2 Key Laboratory for Matter Microstructure and Function of Hunan Province, Key Laboratory of Low-dimensional Quantum Structures and Quantum Control, School of Physics and Electronics, Hunan Normal University, Changsha, 410081 China; 3 Laboratory of Structural Biology, Tsinghua University, Beijing, 100084 China; 4 State Key Laboratory of Medicinal Chemical Biology, Nankai University, Tianjin, 300353 China; 5 ImmVira Co., Ltd, Silver Star Hi-tech Industrial Park, Longhua District, Shenzhen, 518116 China

**Dear Editor,**

*Herpesviridae* is a large family of double-stranded DNA (dsDNA) viruses that cause a variety of human diseases ranging from cold sores and chicken pox to congenital defects, blindness and cancer (Chayavichitsilp et al., 2009; Wang et al., 2018). In the past 70 years, substantial advances in our knowledge of the molecular biology of herpesviruses have led to insights into disease pathogenesis and management. However, the mechanism for capsid assembly that requires the ordered packing of about 4,000 protein subunits into the hexons, pentons and triplexes remains elusive. It is still a puzzle how initially identical subunits adopt both hexameric and pentameric conformations in the capsid and select the correct locations needed to form closed shells of the proper size. Biochemical and genetic studies have shown that the portal is involved in initiation of capsid assembly (Newcomb et al., 2005) and functions akin to a DNA-sensor coupling genome-packaging achieved by a genome-packaging machinery—“terminase complex” (Chen et al., 2020; Yunxiang Yang, 2020) with icosahedral capsid maturation (Lokareddy et al., 2017). Structural investigations of the herpesvirus portal have proven challenging due to the small size of this dodecamer, which accounts for less than 1% of the total mass of the capsid protein layer and the technical difficulties involved in resolving non-icosahedral components of such large icosahedral viruses (diameter is ~1,250 Å). Efforts of many investigators over two decades have made to reconstruct the cryo-electron microscopy (cryo-EM) structure of herpesvirus portal vertex and more recently near-atomic structures of two herpesvirus (herpes simplex virus type 1 (HSV-1) and Kaposi’s sarcoma-associated herpesvirus (KSHV)) portal vertices were reported (McElwee et al., 2018; Gong et al., 2019; Liu et al., 2019).

Here we show asymmetric reconstructions of herpes simplex virus 2 (HSV-2) B- (containing scaffold proteins inside, putative capsid assembly intermediate), C- (DNA-filled capsid, derived from the enveloped virion by using detergent treatment to remove viral membrane proteins and outer tegument proteins) and virion-capsids (enveloped intact virion) at 8.1, 9.0 and 10.2 Å respectively (Figs. S1–3). The asymmetric reconstructions reveal a unique portal vertex in the context of the icosahedral capsid, including 1 portal, 11 pentons, 3 types of hexons (P, peripentonal; E, edge; C, center) with the hexameric rings formed by VP26s, 320 triplexes (Ta-Tf) and 12 pentagram-shaped capsid vertex specific component (CVSC) densities (composed of a UL17 monomer, a UL25 dimer and a UL36 dimer, presence in C- and virion-capsids) (Fig. 1A–C). The validation of our asymmetric reconstructions is based on previous studies in which several herpesvirus capsid structures were determined at near-atomic resolution by cryo-EM and icosahedral averaging (Yu et al., 2017; Jialing Wang, 2018; Yuan et al., 2018). Such averaging ignores the fact that one pentameric vertex is occupied by the portal vertex in the capsid and eliminates the density for any non-icosahedral structural features.

**Figure 1:**
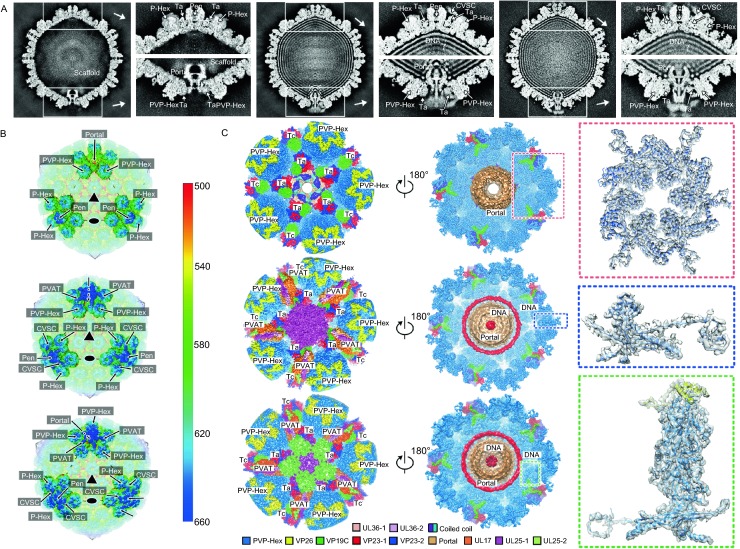

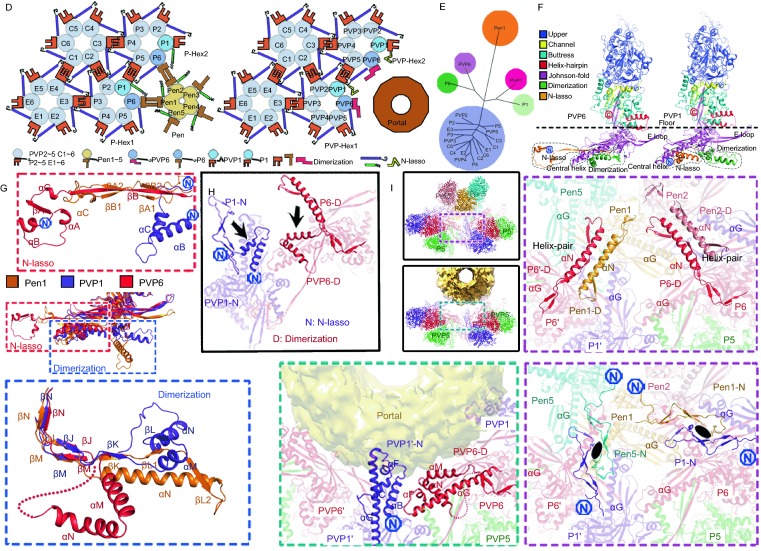
**Overall structures of the portal vertex and organization of VP5**. (A) Central slices of asymmetric reconstructions of HSV-2 B-, C- and virion-capsids. The inserts show the densities of the penton and portal vertices. P-Hex, PVP-Hex, Ta, and Pen denote peripentonal hexons, portal vertex periportal hexons, the triplex A and pentons, respectively. (B) Locations of portal and penton vertex components on the radially colored asymmetric reconstructions of B-, C- and virion-capsids. The pseudo three-fold and two-fold icosahedral symmetry axes are marked as triangles and ellipses, respectively. (C) Cryo-EM maps of B-, C-capsid and viron portal vertices. The insets show the density maps and related atomic models, which illustrate polypeptide backbone and many bulky side chains features. (D) Schematic diagram of the binding mode of different capsomers. Domains with significant conformational differences (Dimerization and N-lasso domains) are highlighted. (E) Structure-based phylogenetic tree of 22 types of VP5 present in the capsid. (F) Ribbon diagram of PVP1 and PVP6. N and C termini are labeled, and major conformational changes are marked with dashed lines. (G) A superposition of the floor of three representative VP5s (Pen1, PVP1, and PVP6). Three types of the N-lasso and dimerization domains are enlarged in red and blue insets, respectively. (H) A superposition of P1 and P6 structures onto PVP1 and PVP6 structures, highlighting the conformational retractions of the PVP1 N-lasso and PVP6 dimerization domains. (I) Comparisons of the intercapsomer interactions between penton and P-Hexs and between portal and PVP-Hexs. The insets show the conformational changes at the vertices. Five helix-pairs and a set of five quasi-equivalent two-fold interactions contributing to the majority of the interactions of P-Hex and penton are replaced by five sets of two four-helix bundles in the portal vertex. The quasi-equivalent two folds axes are marked with ellipses. D and N denote Dimerization and N-lasso domains, respectively

Central slices through the three asymmetric reconstructions show the features of the portal vertex (Fig. 1A). The presence of scaffold proteins, some of which possibly associate with the portal, is observed in the B-capsid. Despite distinct organizations of the CVSC at the portal and penton vertices, the packaged dsDNA is visible in the C- and virion-capsids (Fig. 1A). Radially colored representations of the three reconstructions reveal the dodecameric portal situated beneath one of the pentameric vertices; but, the density surrounding the portal displays a five-fold symmetrical assembly, highlighting the symmetry-mismatch structural features (Fig. 1B). In contrast to B-capsid, small tail-like density, previously named Portal Vertex Associated Tegument (PVAT) (Schmid et al., 2012; McElwee et al., 2018), extends outwards from the portal vertex in the C- and virion-capsids (Fig. 1B), suggesting a possible role in genome retention and release. When five-fold symmetry was imposed, the resolution of local reconstructions for the portal vertex in B-, C- and virion-capsids was improved to 4.05, 4.50 and 5.36 Å, respectively, albeit that the portal was five-fold averaged (Figs. 1C and S3). The resulting density maps clearly reveal the polypeptide backbone, many bulky side chains, and atomic models of the portal vertex associated P-hexon (PVP-Hex), Ta and PVAT (not in B-capsid) from three capsids were built (Figs. 1C and S4).

Apart from the lack of the CVSC, PVAT and genome in the B-capsid and conformational differences on the portal as well as PVAT, the structures of the three capsids are indistinguishable (Fig. S5). While surrounding the portal, PVP-Hex also contacts neighboring hexons, indicative of that PVP-Hex has to adopt conformational changes to allow three distinct interactions: one each with neighboring hexons, the portal and themselves (Fig. 1D). As expected, structure-based phylogenetic analysis of 22 types of VP5 (16 VP5s from an asymmetric unit plus 6 VP5s from PVP-Hex; subunits from C-Hex, P-Hex, E-Hex, PVP-Hex and Pen denoted as C1-C6, P1-P6, E1-E6, PVP1-PVP6 and Pen1-Pen5, respectively) shows 4 subgroups: P1, P6, PVP1 and PVP6 linking typical hexon- and penton-type VP5s (Fig. 1E). Similar to the other types (Yuan et al., 2018), each VP5 from PVP-Hex also folds into seven domains: upper, buttress, helix-hairpin, channel, Johnson fold, dimerization and N-lasso, which constitute the protrusion and floor (Fig. 1F). The domains of N-lasso and dimerization dominating extensive intercapsomer interactions at the inner capsid surface, exhibit three fundamental configurations (Figs. 1G–I and S6). Two of these configurations have been characterized previously (Yuan et al., 2018). A ~45-Å-long extended lasso structure and a shorter β-hairpin for the N-lasso, while a ~40-Å-long helix (αN) and two-short-perpendicular helices (αM and αN) for the dimerization domain have been noted (Fig. 1G). The third types of the configurations of the N-lasso and dimerization domains have now been identified in PVP1 and PVP6, respectively. The N-lasso in PVP1 refolds from a β-strand-rich structure into two helices, resulting in a loss of its lassoing ability, whilst PVP6 dimerization domain moves from nearby the E-loop to the central helix abrogating the two-fold interactions (Fig. 1G–I). In comparison to P1 and P6, both PVP1 N-lasso and PVP6 dimerization domains retract from the distal region by ~ 30 Å leaving more space for the slightly bigger portal (Fig. 1H and 1I). In the penton vertex, a set of five helix-pairs comprising two long αN helices from the dimerization domain of Pen-VP5 and P6 and a set of five quasi-equivalent two-fold interactions formed by β-hairpins from the N-lasso domain of Pen-VP5 and P1, contribute to the majority of the interactions of P-Hex and the penton (Fig. 1I). However, these interactions are lost due to the replacement of the penton by the portal and the conformational changes of PVP1 N-lasso and PVP6 dimerization in the portal vertex. Instead, five sets of two four-helix bundles formed by PVP1 N-lasso and PVP6 dimerization plus two central helices cluster together and associate with the portal, underpinning the symmetry mismatched structural features (Fig. 1I).

The triplex consists of two copies of VP23 (VP23-1, red color; VP23-2, blue color) and one copy of VP19C and lies among three adjacent capsomers. Recent studies (Aksyuk et al., 2015; Yuan et al., 2018) have suggested that the triplexes confer a pronounced directionality on the process of the capsid assembly (Fig. 2A). In the penton vertex, Ta-Tc, Tc-Tb, Tb-Td and Td-Te exhibit two-fold symmetry and VP19C from Pen-Ta (referring to the Ta in the penton vertex) is oriented outwards from the penton (Fig. 2A). Interestingly, PV-Ta, (referring to the Ta in the portal vertex) rotates itself counterclockwise by 120°, forcing VP19C to orient itself towards the portal and breaking the two-fold symmetry of Ta-Tc in the portal vertex (Fig. 2A and 2B). Specifically, PV-Ta undergoes two steps of rotations: 1) counterclockwise by 120° rotation along Z axis; 2) 5° rotation along X axis, enhancing interactions with neighboring subunits in the process (Fig. 2B). Perhaps correlated with the presentence of the portal, the 120° rotation of PV-Ta facilities VP23-1 in building new connections with PVP6. Compared to Pen1, rotations of the central helix and E-loop of PVP6 further forces an 5° rotation of PV-Ta, leading to maximization of interactions with PVP6 and PVP1 (Fig. 2C). Interestingly a novel network of subunit contacts is observed between the portal and PV-Ta and varies each other in three capsids (Figs. 2D and S7). Lying on the portal head, a novel five-fold symmetrical assembly comprising five copies of a well-resolved two-helix coiled coil (presumably being the fragment from the portal or pUL36) shows close contacts with PV-Ta (Fig. 2D and 2E).

**Figure 2:**
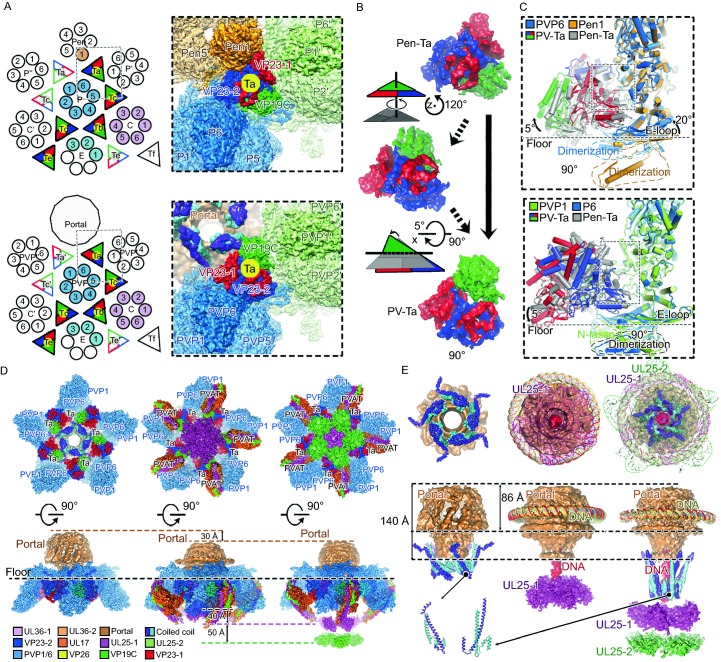
**Rotation of the triplex A and structures features of the portal and PVAT**. (A) Schematic representations of two types of the asymmetric unit (shaded) of the capsid. Extra copies of triplexes and VP5s from adjacent asymmetric units are shown to depict the microenvironments of the penton and portal vertices. The triangle filled with red, blue and green represents the heterotrimeric nature of a triplex. Inserts show the densities of Pen-Ta and PV-Ta with their surrounding subunits. (B) The counterclockwise rotation of PV-Ta. PV-Ta undergoes a two-step rotation. Step 1: counterclockwise 120° rotation along z axis. Step 2: 5° rotation along x axis. (C) Superimpositions of (Pen-Ta)-PEN1 onto (PV-Ta)-PVP6 (top) and (Pen-Ta)-P6 onto (PV-Ta)-PVP1 (bottom). The major conformational changes are marked by dash lines. Notable rotations are labeled. (D) Comparisons of the local microenvironment of the portal vertex from asymmetric reconstructions of B- (left), C- (middle) and virion-capsid (right). (E) Structural comparisons of the portal and PVAT from B-, C- and virion-capsids. The two layers of the UL25 C-domain are set with 90% transparency in the top view. The color scheme is same as in Fig. 1C and the density for dsDNA is colored in red

The portal vertex of the capsid is a hub for protein-protein interactions that are important for capsid assembly and genome packaging. A comparison of asymmetric reconstructions of the portal vertex of the three capsids reveals notable differences in the position and morphology of the portal and PVAT (Fig. 2D). Strikingly, the portal in the B-capsid situates inwards by ~30 Å. It exposes itself by lacking the PVAT and forms a basin at the portal vertex, suggesting a ready-state for docking of terminase complexes (Yunxiang Yang, 2020) and genome packaging, when compared to those in the C- or virion-capsids (Fig. 2D). Distinct from the virion-capsid, a bulk of obvious densities presumably emanating from dsDNA beneath the portal is observed (Fig. S8), however some exterior densities, corresponding to five copies of coiled coils and five copies of the UL25 C domain, become disordered or lost in the C-capsid, consistent with previous observations of the CVSC of HSV-2 C-capsid (Jialing Wang, 2018) (Fig. 2D). These might result from the lack of protection offered by tegument proteins/envelope, indicative of the vulnerability of the role in the retention of viral genome of isolated C-capsids *in vitro*, reflecting purified C-capsids are metastable and tend to release their genome. In contrast, five 10-nm-long coiled coils in the virion-capsid exhibit altered configurations to the counterparts in B-capsid to recruit the PVAT layers (UL25 C-domain), ensuring the retention of DNA (Fig. 2E).

In tailed bacteriophages, the procapsid portal changes conformation to a mature virion state during the DNA packaging process (Lee and Guo, 1995; Lokareddy et al., 2017). More sophisticatedly, the portal vertex in herpesviruses alters its components and morphology to trigger the switches from a pre-DNA-packaging intermediate to a DNA-packaged mature state and further to a pre-DNA-releasing metastability, in which the portal together with five copies of coiled coils presumably acts as an adaptor-sensor for genome bidirectional delivery, transmitting signals between the internal genome and exterior PVAT layers during genome packaging (Yunxiang Yang, 2020) or releasing. A detailed understanding of the pathway of herpesvirus capsid maturation and the availability of a rich variety of herpesvirus mutants, including those that block DNA packaging or retention or capsid maturation, could open up new avenues for more precise, structure-based mutagenesis approaches for development of better antiviral drugs for treating infections caused by herpesviruses.

## Electronic supplementary material

Below is the link to the electronic supplementary material.

Supplementary material 1 (PDF 2805 kb)
