# Supplementary material for: Structures of the portal vertex reveal essential protein-protein interactions for Herpesvirus assembly and maturation
Source: Protein Cell. 2020 Apr 13;11(5):366–73. doi: 10.1007/s13238-020-00711-z (PMC7196605; doi:10.1007/s13238-020-00711-z)
Supplement: Supplementary file 1 — Supplementary material 1 (PDF 2805 kb) [file 13238_2020_711_MOESM1_ESM.pdf]

Supplementary information

Structures of the portal vertex reveal essential protein-protein interactions for Herpesvirus assembly and maturation

Nan Wang[1$], Wenyuan Chen[2$], Ling Zhu[1$], Dongjie Zhu[1], Rui Feng[1], Jialing Wang[1], Bin Zhu[2], Xinzheng Zhang[1], Xiaoqing Chen[5], Xianjie Liu[5], Runbin Yan[5], Dongyao Ni[5], Grace Guoying Zhou[5], Hongrong Liu[2]*, Zihe Rao[1,3,4]* and Xiangxi Wang[1]*

This file includes:
Methods
Figures S1-S8
Table S1

ONLINE METHODS

Virus culture and purification

HSV-2 virus genotype *MS* was propagated in Vero cells at a multiplicity of infection (MOI) of 0.2 at 37 °C. Capsid and virion production and purification have been described previously (Jialing Wang, 2018; Wang et al., 2012; Wang et al., 2015; Yuan et al., 2018).

Negative stain

Purified B-, C-capsids and virions were diluted in PBS (pH 7.4) and concentrated to a suitable concentration. A 3-μL aliquot of purified capsids was added to a freshly glow-discharged grid, washed twice with PBS, and strained with phosphotungstic acid (pH 7). All samples were checked on a 120 KV electron microscope.

Cryo-EM and data collection

For cryo-grid preparation of three types of capsids, a 3-μL aliquot of capsids was applied to a fresh glow-discharged copper grid. Grids were blotted for 3.5s in 80% relative humidity for plunge-freezing (Vitrobot; FEI) in liquid ethane. Titan Krios microscope with Falcon3 detector was used to collect data. Movies were recorded as 25 frames at a total does of 25 $e^{-}Å^{-2}$ and defocus range from 0.8 to 2.3 μm. The magnification under these conditions was 59,000 x, which yielded a final pixel size of 1.38 Å.

Data Processing

A total of 5,567 micrographs for B-capsid; 8,413 micrographs for C-capsid and 12,730 micrographs for virion were recorded. Frames 3–22 were used and corrected for beam-induced drift by aligning and averaging the individual frames of each movie using MOTIONCORR2 (Zheng et al., 2017). The contrast transfer function (CTF) parameters for drift corrected micrographs were estimated by Gctf (Zhang, 2016). Particles were picked manually by EMAN2 package. A total of 41,956 particles from 4,600 micrographs for B-capsid, 56,901 particles from 7,023 micrographs for C-capsid and 29,221 particles from 12,700 micrographs for virion were selected for the two-dimensional alignment and three-dimensional reconstruction using the block-based reconstruction (Wang et al., 2019; Wang et al., 2017a; Yuan et al., 2018; Zhu et al., 2018a). Briefly, in our case, the parameters of the icosahedral orientation and the center of each particle determined by Relion were used to guide extraction of components of the blocks (~50% bigger than the penton) to refine and reconstruct separately with their local mean defoci. In each boxed cryo-EM image, there were 12 icosahedral-symmetry related copies (440,844 blocks in total for B-capsids; 682,812 blocks in total for C-capsids and 191,856 blocks in total for virion-capsids) for the blocks. After knowing the rotation and translation parameters of the virus, the distance *d* between the center of one copy in the 3D virus and the center of the virus along the *Z* axis (parallel to the incident electron beam) was calculated to solve the gradient in defocus through the capsid. This local defocus of each copy instead of the uniform defocus obtained by fitting was used to reconstruct the vertices. After

refinement and reconstruction of the blocks in Relion (Scheres, 2012), two cycles of 3D classification with no-alignment were used to enrich the portal vertices from the blocks for B-, C- and virion-capsids. For the B-capsid, two cycles of 50 iterations of 3D classifications using a ball with a diameter of 250 Å as a mask were performed. ~80% of blocks which are not likely to contain the portal vertices could be separated from the first cycle of 3D classification and the rest (~20%) blocks from one major class were then used for the second cycle of 3D classification with 3 classes. After that, 22,158 blocks (~5%) from one class exhibiting the portal features were subjected to one cycle of automatic 3D refinement with C5 symmetry, which yielded a reconstruction for the B-capsid portal vertex at 4.05 Å resolution. For the C- and virion-capsids, we applied a similar strategy to that of the B-capsids, except a column mask with a diameter of 120 Å and height of 300 Å was used. ~35,698 blocks (5.2%) and ~12,001 blocks (6.2%) with the portal features were enriched and subjected to 3D refinement with C5 symmetry, leading to a 4.5 Å and 5.36 Å resolution density map for the C- and virion-capsid portal vertex, respectively.

All Euler angles parameters of selected blocks were applied to the bin2 particles. For an icosahedral virus, each vertex has a C5 symmetry. The 5 equivalent orientations of the vertex can be obtained by common-line algorithm (Liu and Cheng, 2015; Wang et al., 2017b; Zhu et al., 2018b). We assumed that the portal structure has a fixed orientation related to the five-fold symmetric vertex. The correct orientation of the portal can be determined by searching for the 5 equivalent orientations of the vertex. We randomly chose one of the 5 equivalent orientations of its vertex as the initial orientation for each raw particle image. Then the following 3 steps were performed to determine the exact orientation information. (1) We reconstructed a full particle structure (3D model) using the raw particle images. (2) For each image, we projected the 3D model to generate 5 projection images according to the 5 equivalent orientations of the vertex. Meanwhile, we boxed out images (160×160 pixel) around the vertex of the portal to calculate the correlation between corresponding original image and projection images. We searched the best matched projection image with the original images. (3) We performed iterations of step (1) and (2) until the orientations of individual original images were stable and no further improvement of the portal structure could be obtained. For the B-capsid, we performed a reconstruction with C1 symmetry when the iteration came to the 20th cycle. We obtained a portal structure with a C12 symmetry. The C- and virion-capsids portal structures with a C12 symmetry were observed at the 34th cycle of iteration. Finally, asymmetric reconstructions of the B-, C- and virion-capsids were obtained at 8.07 Å, 9.03 Å and 10.19 Å, respectively. The overall resolution for all reconstructions were evaluated based on gold-standard Fourier shell correlation (FSC) = 0.143 criterion (Scheres, 2012).

Model building and refinement

The structures of P-Hex and triplexes of HSV-2 B-capsid (PDB code: 5ZAP) (Yuan et al., 2018) and P-Hex, triplexes and CVSC of HSV-2 C-capsid (PDB code: 5ZZ8)

(Jialing Wang, 2018) were initially fitted into the five-fold averaged EM maps of the B-, C-capsid and viron portal vertices, respectively, with CHIMERA and further corrected manually by real-space refinement in COOT. The poly-alanine model of the five coiled coils was built *de novo* into density using COOT. These models were further refined by positional and B-factor refinement in real space using Phenix and rebuilt in COOT iteratively. Only the coordinates were refined keeping the maps constant. The data set and refinement statistics are summarized in Table S1.

Data availability

The atomic coordinates of the portal vertex from HSV-2 B-, C- and virion-capsids have been submitted to Protein Data Bank with accession numbers PDB: 6M6I, 6M6H and 6M6G, respectively. Cryo-EM density maps of asymmetrically reconstructed HSV-2 B-, C- and virion-capsids and five-fold symmetrically reconstructed portal vertices from HSV-2 B-, C- and virion-capsids have been deposited with the Electron Microscopy Data Bank: EMD-30120, EMD-30122, EMD-30121, EMD-30125, EMD-30124, and EMD-30123 respectively. The data that support the findings of this study are available from the corresponding author upon request.

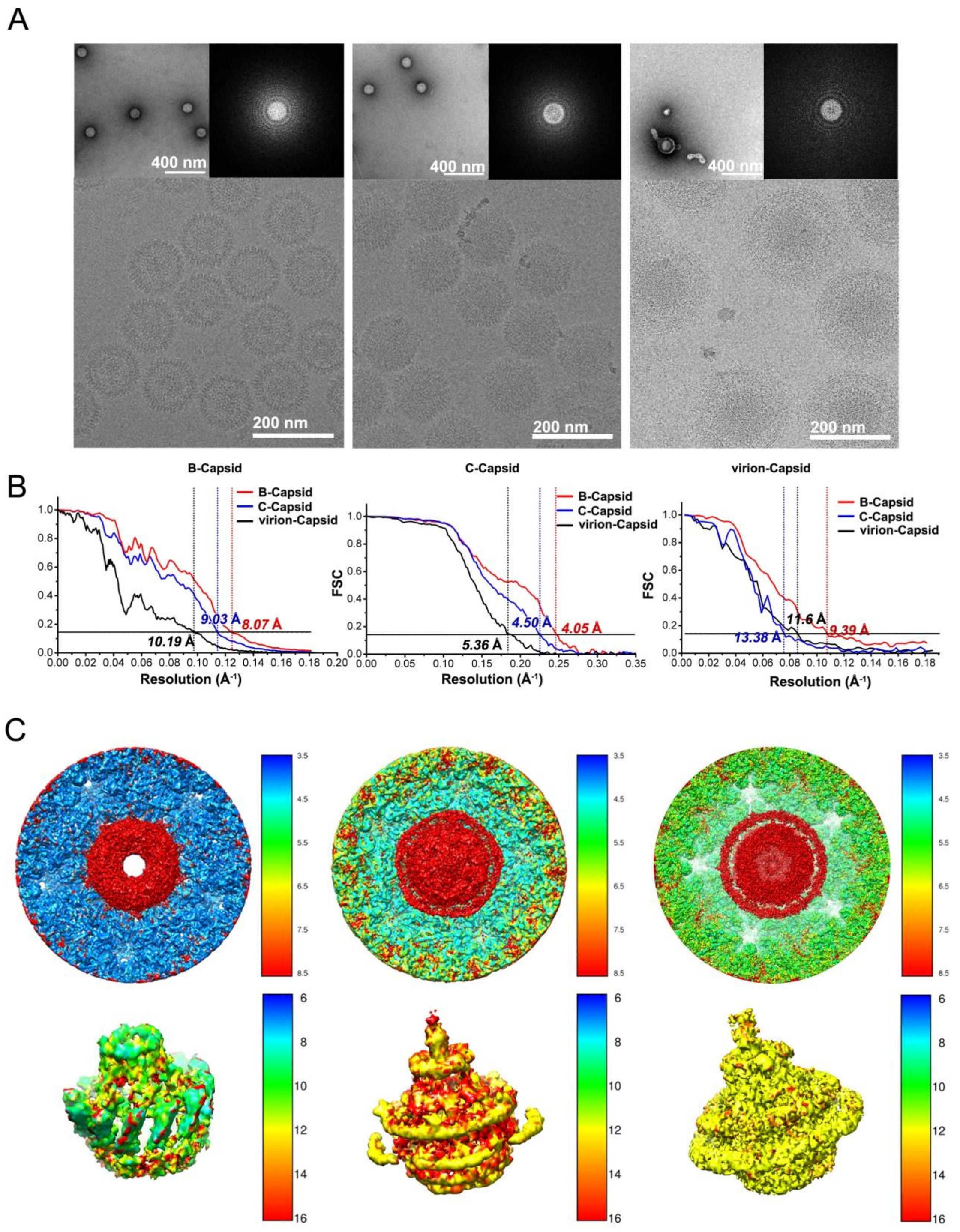

Fig. S1 Resolution evaluation of the cryo-EM map.

(A) Negative stained images (top left), cryo-EM images (bottom), and the Fourier transformation (top right). (B) The gold-standard FSC curves. FSC plots of the asymmetric reconstructions (left), the portal vertices with C5 symmetry (middle) and the portal with C1 symmetry (right) based on the FSC=0.143 criterion are shown. (C), The final maps were analyzed by ResMap (Kucukelbir et al., 2014). The reconstructions of the portal vertices from B- (top left), C- (top middle) and virion-capsids (top right) with C5 symmetry and the asymmetric reconstructions of the

portals from B- (bottom left), C- (bottom middle) and virion-capsids (bottom right) are shown.

A

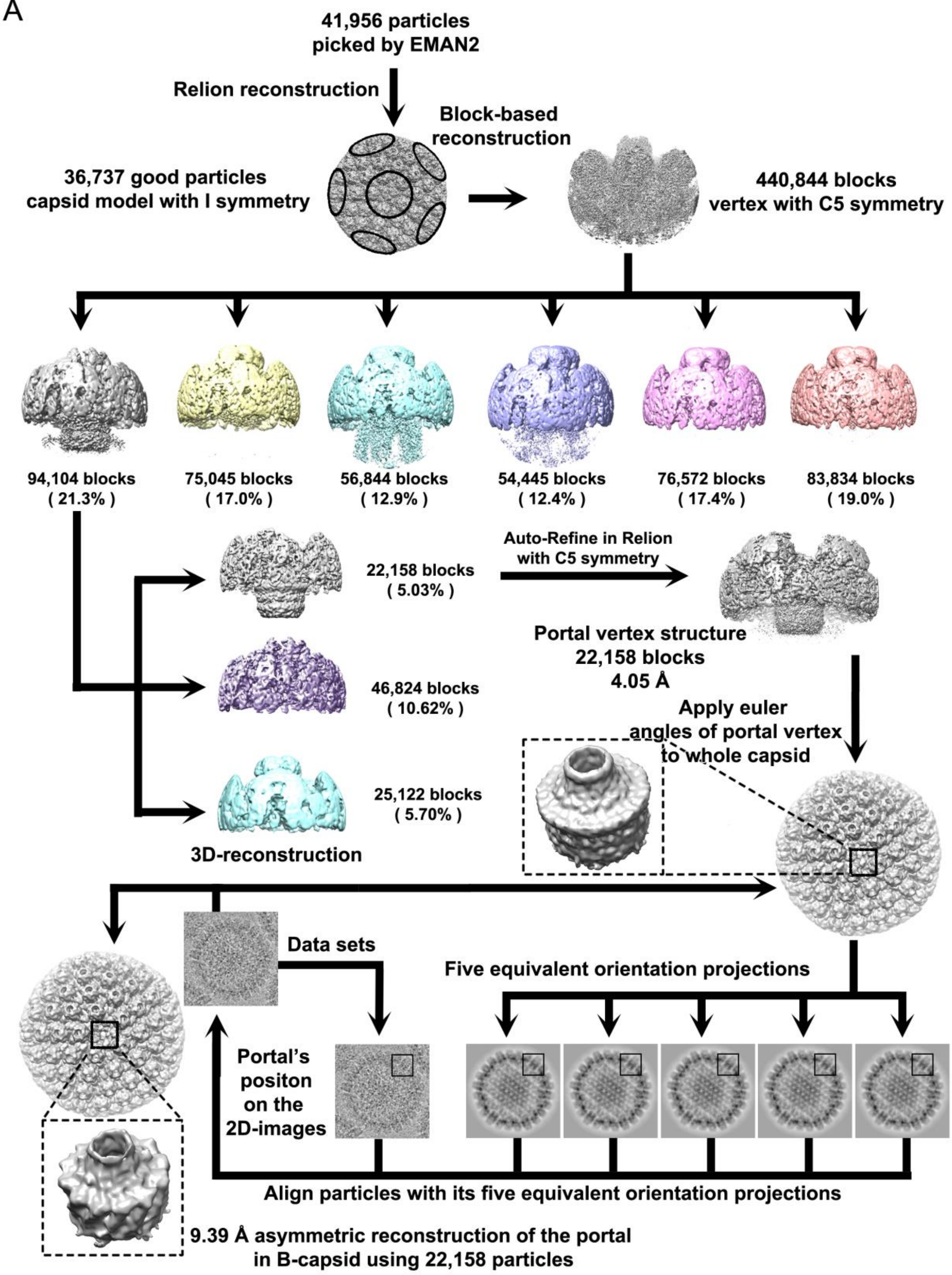

41,956 particles
picked by EMAN2
Relion reconstruction
Block-based
reconstruction
36,737 good particles
capsid model with I symmetry
440,844 blocks
vertex with C5 symmetry
94,104 blocks
(21.3%)
75,045 blocks
(17.0%)
56,844 blocks
(12.9%)
54,445 blocks
(12.4%)
76,572 blocks
(17.4%)
83,834 blocks
(19.0%)
22,158 blocks
(5.03%)
Auto-Refine in Relion
with C5 symmetry
Portal vertex structure
22,158 blocks
4.05 Å
46,824 blocks
(10.62%)
Apply euler
angles of portal vertex
to whole capsid
25,122 blocks
(5.70%)
3D-reconstruction
Data sets
Five equivalent orientation projections
Portal's
positon
on the
2D-images
Align particles with its five equivalent orientation projections
9.39 Å asymmetric reconstruction of the portal
in B-capsid using 22,158 particles

B

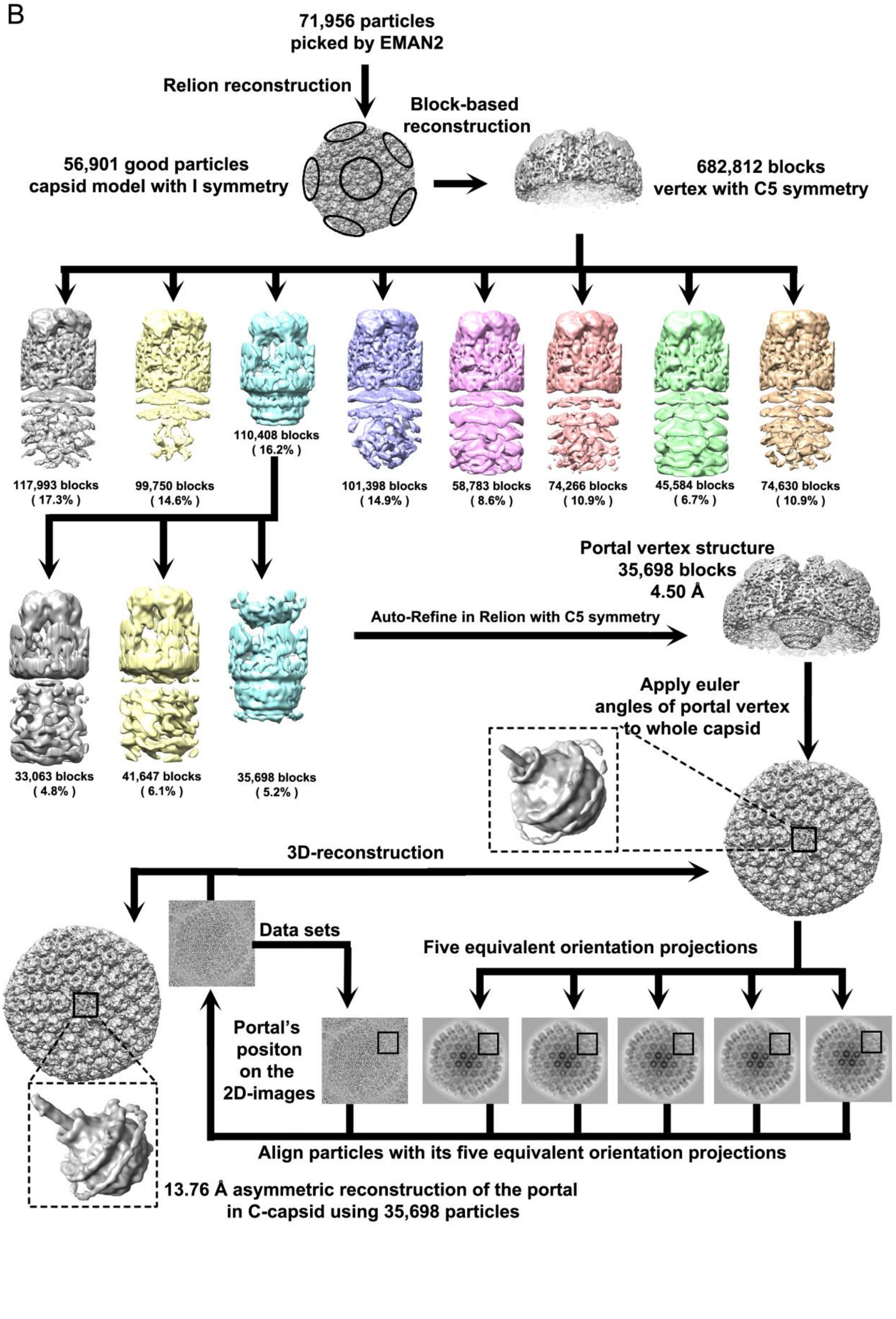

71,956 particles
picked by EMAN2
Relion reconstruction
Block-based
reconstruction
56,901 good particles
capsid model with I symmetry
682,812 blocks
vertex with C5 symmetry
117,993 blocks
(17.3%)
99,750 blocks
(14.6%)
110,408 blocks
(16.2%)
101,398 blocks
(14.9%)
58,783 blocks
(8.6%)
74,266 blocks
(10.9%)
45,584 blocks
(6.7%)
74,630 blocks
(10.9%)
Portal vertex structure
35,698 blocks
4.50 Å
Auto-Refine in Relion with C5 symmetry
33,063 blocks
(4.8%)
41,647 blocks
(6.1%)
35,698 blocks
(5.2%)
Apply euler
angles of portal vertex
to whole capsid
3D-reconstruction
Data sets
Five equivalent orientation projections
Portal's
positon
on the
2D-images
Align particles with its five equivalent orientation projections
13.76 Å asymmetric reconstruction of the portal
in C-capsid using 35,698 particles

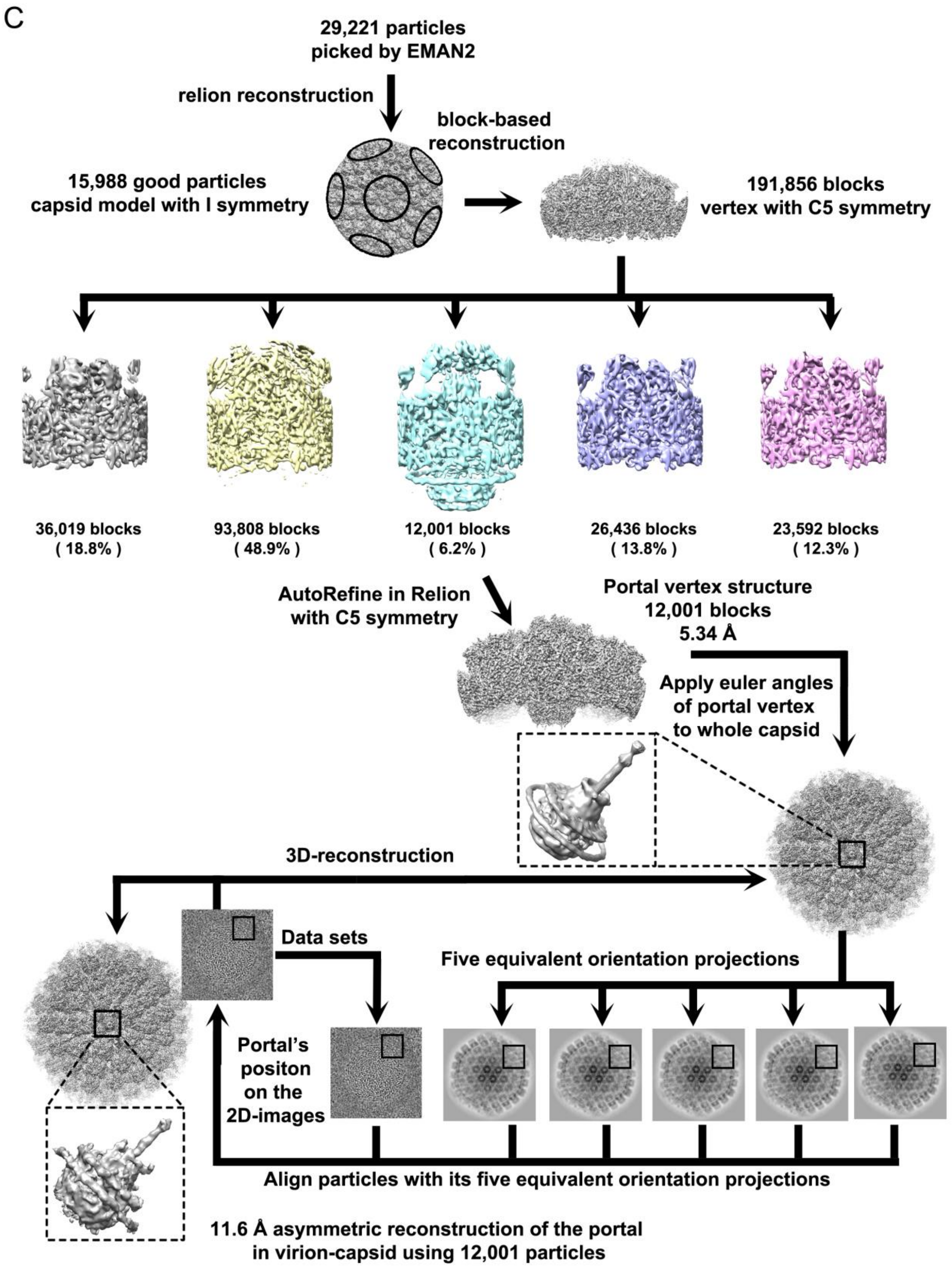

Fig. S2 The flowchart for EM data processing.

Data processing and asymmetric reconstructions of B- (A), C- (B) and virion-capsids (C). Details can be found in Methods.

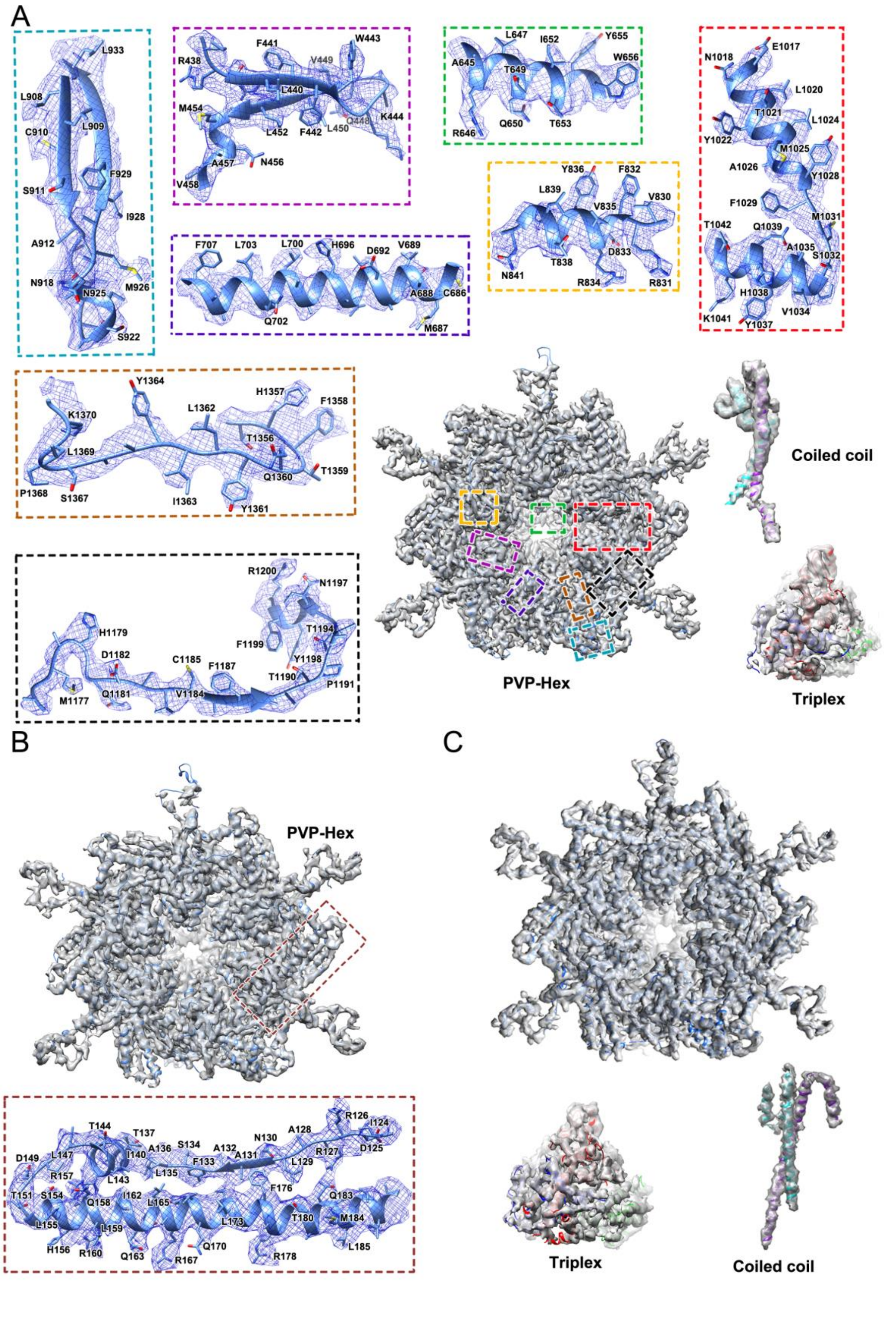

Fig. S3 Density maps and atomic models of the portal vertices from B- (A), C- (B) and virion-capsids (C).

The polypeptide backbones, many bulky side chains, and extensive subunit contacts are revealed clearly by the resulting density maps.

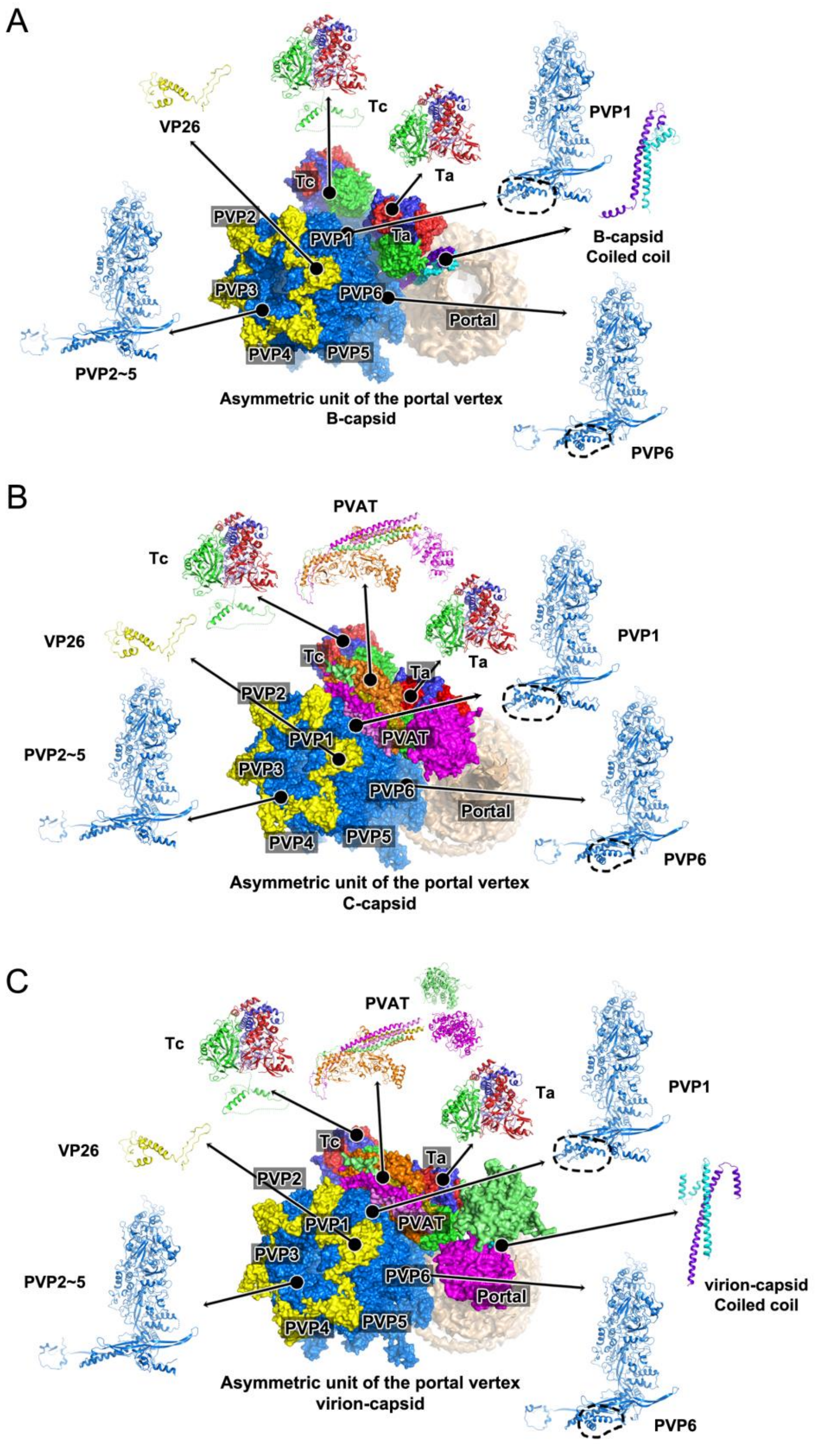

Fig. S4 Ribbon diagram representation of the atomic models of the asymmetric unit of B- (A), C- (B) and virion-capsids (C) portal vertices.

Ribbon diagram representation of the atomic models of the asymmetric unit of B-, C- and virion-capsids portal vertices. The cartoon models show the individual protein and conformers (major conformational changes are marked by dashed lines). The color scheme is same as in Fig. 1C.

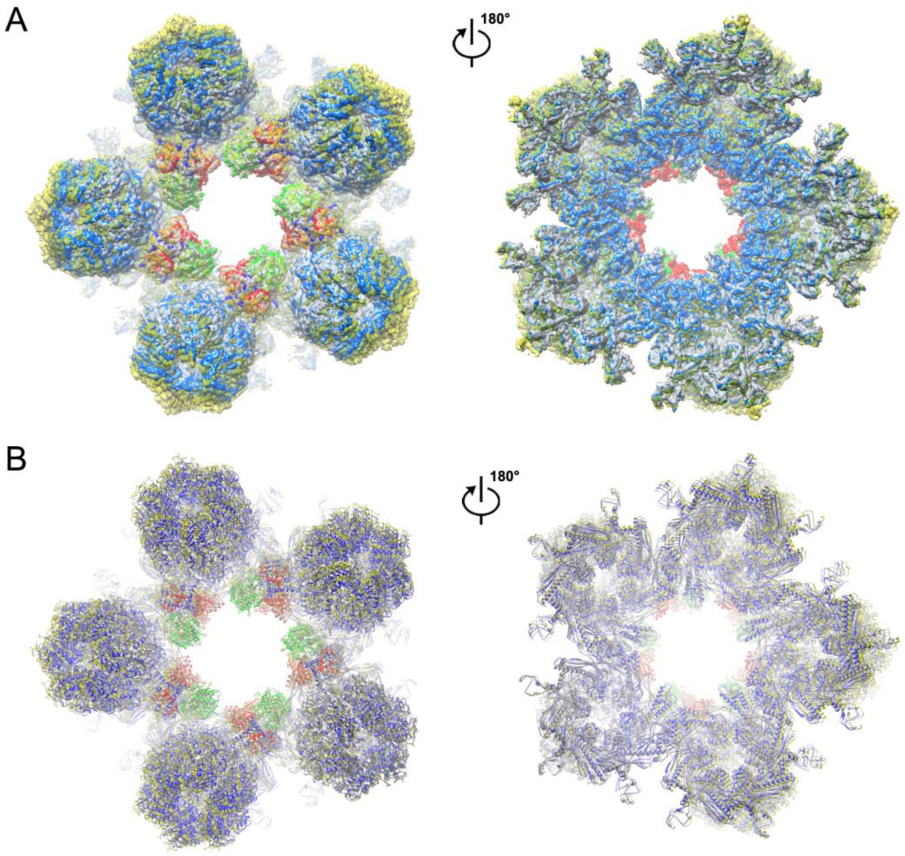

Fig. S5 Structural comparison of the capsid proteins and triplexes in the portal vertices from B-, C- and virion-capsids.

The portal vertices superimposition. Cryo-EM maps (**A**) and atomic models (**B**) of the portal vertices from B- and C-capsids are superposed on the map and atomic model of the portal vertex from the virion-capsid, respectively. The color scheme for the portal vertex from B-capsid is same as in Fig. 2. The portal vertices from C- and virion-capsids are colored in gray and yellow, respectively.

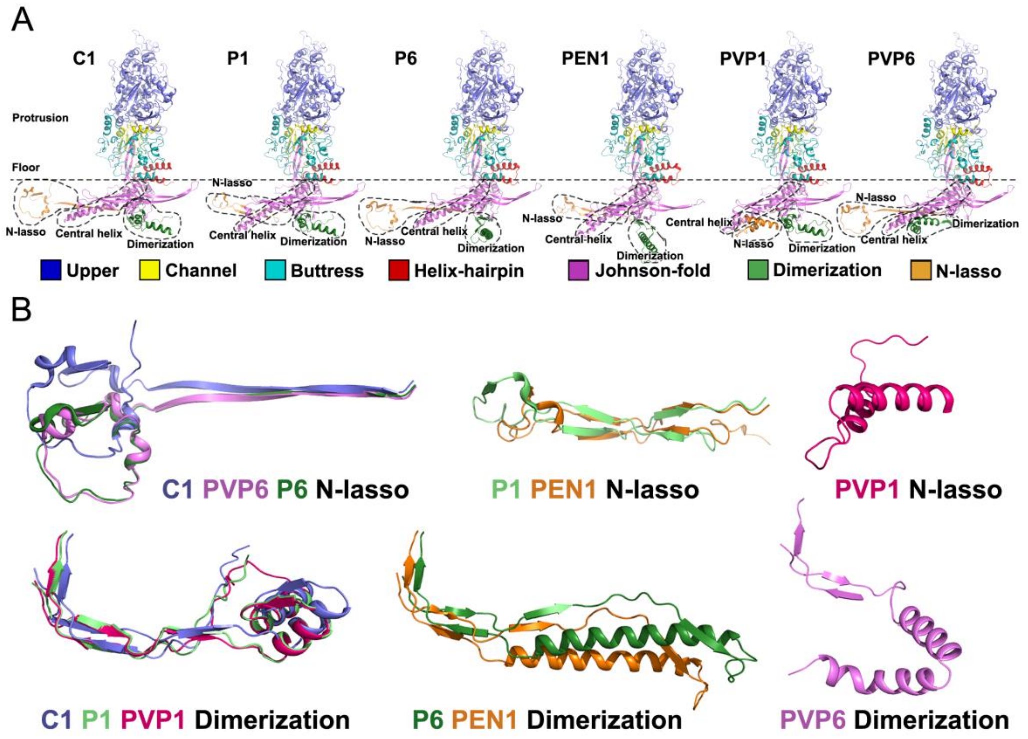

Fig. S6 Fundamental configurations of N-lasso domain and Dimerization domain (A) Ribbon diagram of typical hexon-, penton-, and portal vertex periportal VP5. The major conformational changes are marked with dash lines. The upper and middle sections make up the capsid protrusions. (B) Three fundamental configurations of N-lasso domain (top) and Dimerization domain (bottom) are shown.

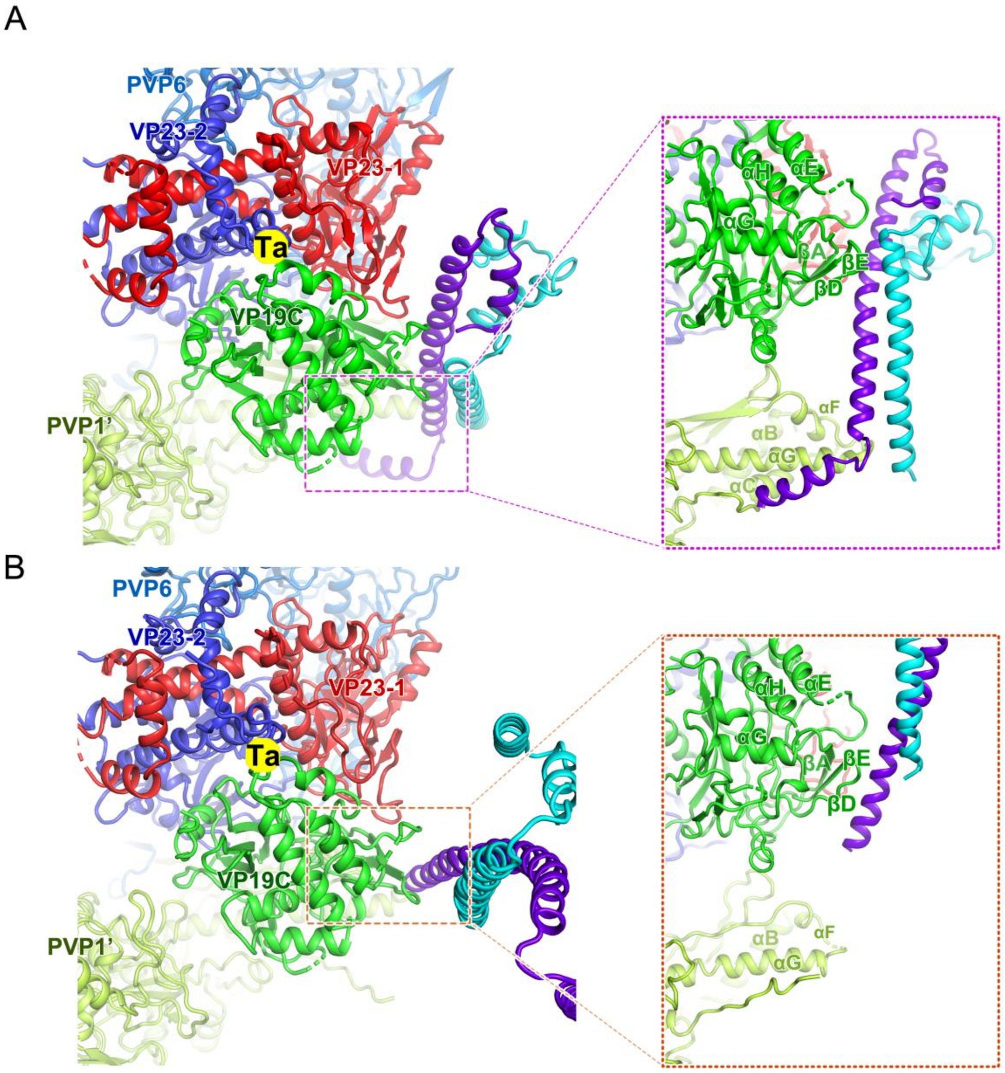

Fig. S7 Interactions between five coiled coils and their neighboring subunits in B-(A) and virion-capsids (B).

Color scheme is same as in Fig. 2C. Insets show the close contacts between the coiled coils and their surrounding subunits.

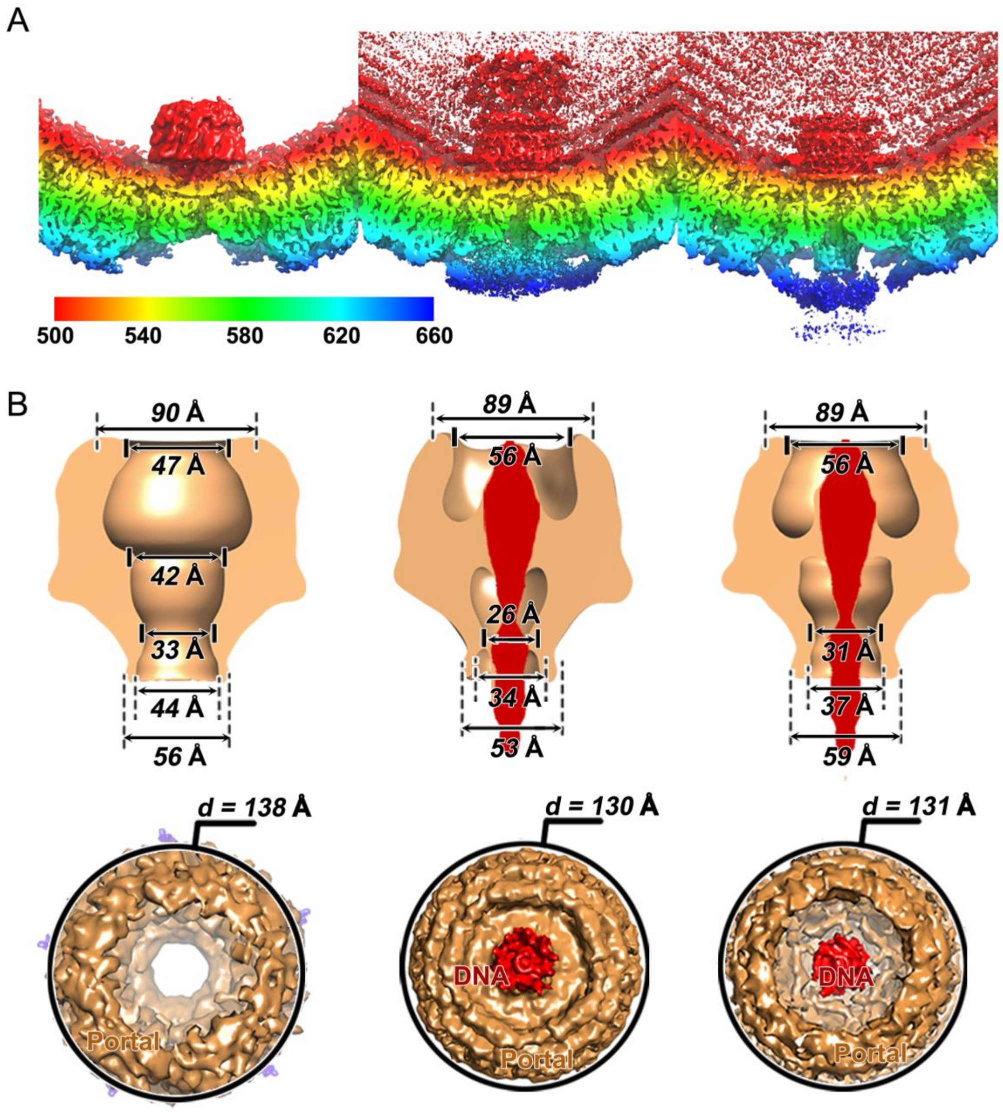

Fig. S8 Structural comparisons of the portal from B-, C- and virion-capsids
(A) Radially colored representations of asymmetric reconstructions of B-, C- and virion-capsids. (B) The inner surface (top) and bottom (bottom) views of the portal from B-, C- and virion-capsids.

Table S1 Cryo-EM imaging, data processing, and refinement statics

	B-capsid	B-PV	C-capsid	C-PV	virion-capsid	virion-PV
**Data collection and processing**						
Magnification	59,000		59,000		59,000	
Voltage (kV)	300		300		300	
Electron exposure (e-/Å^2)	25					
Defocus range (μm)	1.5-2.5					
Pixel size (Å)	1.38		1.38		1.35	
Symmetry imposed	C1	C5	C1	C5	C1	C5
Initial particle image (no.)	41,956		71,956		29,221	
Final particle image (no.)	22,158		35,698		12,001	
Map resolution (Å)						
FSC threshold	0.143					
Map resolution range (Å)	8.07	4.05	9.03	4.50	10.19	5.36
**Refinement**						
Initial model used (PDB code)	N/A					
Model resolution (Å)	N/A					
FSC threshold						
Model resolution range (Å)	N/A					
Model sharpening B factor (Å^2)	N/A					
Model composition						
Non-hydrogen atoms	N/A	72,463	N/A	78,032	N/A	85,524
Protein residues	N/A	9,791	N/A	10,426	N/A	11,546
R.m.s deviations						
Bonds lengths (Å)		0.01		0.01		0.01
Bonds angles (º)		1.06		1.16		10.8
Validation	N/A		N/A		N/A	
Clashscore		7		8		10
Poor rotamers (%)		0.5		0.6		0.9
Ramachandran plot	N/A		N/A		N/A	
Favored (%)		90.05		90.48		90.43
Allowed (%)		9.71		9.09		9.02
Disallowed (%)		0.24		0.43		0.55
